# Relationships Between Training Workload Parameters with Variations in Anaerobic Power and Change of Direction Status in Elite Youth Soccer Players

**DOI:** 10.3390/ijerph17217934

**Published:** 2020-10-29

**Authors:** Hadi Nobari, Luis Felipe Tubagi Polito, Filipe Manuel Clemente, Jorge Pérez-Gómez, Mina Ahmadi, Miguel Ángel Garcia-Gordillo, Ana Filipa Silva, Jose Carmelo Adsuar

**Affiliations:** 1 Department of Exercise Physiology, Faculty of Sport Sciences, University of Isfahan, Isfahan 81746-7344, Iran; minaahd7@gmail.com; 2 Sports Scientist, Sepahan Football Club, Isfahan 81887-78473, Iran; 3 Departamento de Educação Física, Universidade São Judas Tadeu, Av. Vital Brasil, São Paulo 03321-001, Brazil; luis.polito@uol.com.br; 4 Escola Superior Desporto e Lazer, Instituto Politécnico de Viana do Castelo, Rua Escola Industrial Comercial de Nun’Álvares, 4900-347 Viana do Castelo, Portugal; filipe.clemente5@gmail.com (F.M.C.); anafilsilva@gmail.com (A.F.S.); 5 Health, Economy, Motricity, and Education (HEME) Research Group, Faculty of Sport Sciences, University of Extremadura, 10003 Cáceres, Spain; jorgepg100@gmail.com (J.P.-G.); carmelo.adsuar@gmail.com (J.C.A.); 6 Facultad de Administración y Negocios, Universidad Autónoma de Chile, Sede Talca 3467987, Chile; miguelgarciagordillo@gmail.com; 7 Núcleo de Investigação (N2i), Polytechnic Institute of Maia, 4475-690 Maia, Portugal; 8 The Research Centre in Sports Sciences, Health Sciences and Human Development (CIDESD), 5001-801 Vila Real, Portugal

**Keywords:** football, soccer, acceleration, deceleration, training monotony, training strain, starters, nonstarters, in-season, pre-season

## Abstract

The purpose of this study was to test the relationships between training workload (WL) parameters with variations in anaerobic power and change of direction (COD) in under-16 soccer players. Twenty-three elite players under 16 years were daily monitored for their WL across 20 weeks during the competition soccer season. Additionally, players were assessed three times for anthropometric, body composition, COD, and anaerobic power. A correlational analysis between the mean differences between assessments and accumulated WL parameters were conducted. Moreover, a regression analysis was executed to explain the variations in the percentage of change in fitness levels considering the accumulated WL parameters and peak height velocity. The accumulated daily loads during one week showed a large and a moderate correlation with peak power and COD at different periods of the season. Regression analysis showed no significant predictions for COD (*F*_(12, 10)_ = 1.2, *p* = 0.41) prediction, acute load (*F*_(12, 10)_ = 0.63, *p* = 0.78), or chronic load (*F*_(12, 10)_ = 0.59, *p* = 0.81). In conclusion, it may be assumed that the values of the chronic workload and the accumulated training monotony can be used to better explain the physical capacities of young soccer players, suggesting the importance of psychophysiological instruments to identify the effects of the training process in this population.

## 1. Introduction

Soccer is a highly complex sport, which can be defined as an intermittent activity, characterized by the use of aerobic and anaerobic metabolic pathways in order to provide energy during different technical and tactical situations during the game [1].

The aerobic metabolism is involved in low-intensity situations, whereas the anaerobic system is involved in the most significant part of the game, including the high intensity explosive efforts, which requires high levels of power [2,3].

The aerobic capacity is needed to maintain the energy providing during all the match, whereas the anaerobic capacity is fundamental in order to execute the main foundations of the game (explosive efforts, kicks, etc) [4].

So, it is safe to affirm that one of the most important variables for measuring the performance in soccer players is the physical conditioning, which involves both aerobic and anaerobic physical capacities [5] and the change of direction (COD) as well [6].

Despite the fact that aerobic and anaerobic power increases with age, which occurs due to biological development [7], the training process can improve the neural and muscular factors related to these physical capacities in young athletes, including the development of energy supply, hydrogen accumulation, and muscle activation [8].

In order to ensure the development of these qualities all season, there are many aspects that need to be controlled to optimize the gain and avoid the injuries, including: intensity, volume, density, mood states, recovery times [9], external and internal training WL (workload), and parameters obtained from this like the acute (AWL), chronic (CWL), acute: chronic workload ratio (ACWLR), training monotony (TM), and training strain (TS). These last five parameters can be obtained from the ratings of perceived exertion (RPE) of the training session [10].

The point is, how much effort is needed to change the physical capacities? To examine this, Bannister and colleagues [11] established a statistical model to explain how the athlete responds to a given training process. According to this model, there are two different training effects: negative (fatigue) and positive (fitness), and the result of the training is the difference between these two effects.

Some studies have been conducted analyzing the possible associations between WL and the changes in physical qualities. Brink and colleagues investigated the relation among training load, recovery, and performance in a monthly interval shuttle run test and did not find any relationship among RPE and total quality recovery scores with the performance (more related to the duration and game play in the week before the test). Other trials were found in adult soccer players [12,13] and in other sports, like rugby [14].

Due to the popularity of soccer in adolescence and the creation of several young championships for this public, some studies have been published analyzing the relationships among different performance parameters. However, there is no found scientific literature analyzing the relationship among the anaerobic power, the COD performance, and the total WL in under-16 soccer players, which justifies this research. Furthermore, it is important to highlight that understanding the process of these relationships can inform the fitness coaches, providing for them important knowledge in order to organize the training program. Therefore, the aim of this study was to analyze the relationships between training WL parameters with variations in anaerobic power and COD in under-16 soccer players.

## 2. Materials and Methods

### 2.1. Participants

Twenty-three elite soccer players U16 Iranian were evaluated. The subjects’ maturity offset were 1.85 ± 0.30 years; this means that they had passed the peak height velocity (PHV). The positions of the soccer players were defenders (n = 9), midfielders (n = 6), wingers (n = 4), and forwards (n = 4). Goalkeepers were not evaluated in the study due to physiological differences in training and competition. Inclusion criteria for this study were as follows; (i) At least 90% of the in-season were trained in the study; (ii) players were not injured in the time frame in the study; (iii) players should not be cross-training within the time frame in the study; and (iv) the number of training sessions for players who did not participate in the weekly competition was adjusted with another session (i.e., high-intensity interval training or small side game). After receiving information about the study, all participants, together with the parents’ consent, signed the consent form to participate in the study. This study started after the approval of the ethical code IR.UI.REC.1397.181 by the University of Isfahan, and in compliance with the declaration of Helsinki for human subjects.

### 2.2. Experimental Approach to the Problem

**Figure 1:**
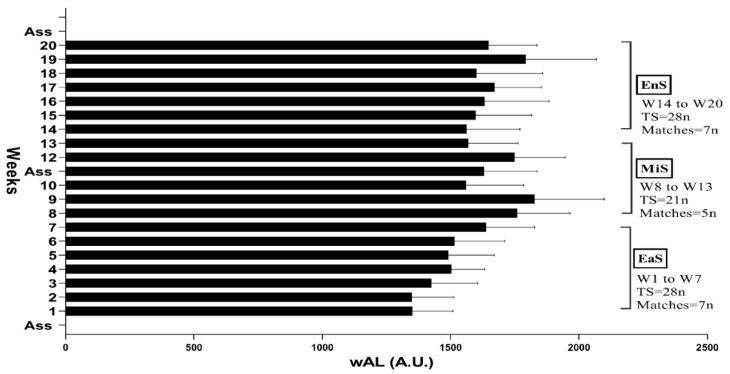
Research outline of the weekly monitoring on training and match load and assessed sessions during the competition season. EaS (early-season = before league for first study and W1 to W7 for the second study); MiS (mid-season = mid league for fist study and W8 to W13 for the second study); and EnS (end-season = after league for fist study and W14 to W20 for the second study); wAL = weekly acute workload; W = Week; TS = Training sessions; ASS = Assessments, and A.U. =Arbitrary unit.

This study includes; (1) studying the cohort along with monitoring the daily workload for 20 weeks in the competition season: early-season (EaS) weeks (w) W1 to W7; mid-season (MiS) W8 to W13; and end-season (EnS) W14 to W20 and (2) a semi-experimental study and 3 stages of evaluation; the first stage of the evaluation took place in the last week of August (EaS = before league); the second stage of the evaluation took place in the third week of November (MiS = mid league); and the third stage of the evaluation was performed in the first week of February (EnS = after league), Figure 1. The number of RPE with the training time session was used to calculated WL [15,16]. Then, AWL, CWL, ACWLR, TM, and TS were obtained from WL. The stages of the subjects’ assessments were as follows: day 1, assessments of anthropometric and body composition (height, sitting height, body mass, body fat, and maturity); day 2, COD with the modified 505 test [17]; and day 3, the anaerobic power were assessments with Running-Based Anaerobic Sprint Test (RAST). All tests were performed at the same time and at the same temperature in the indoor track using a thermometer as the recommendations given for standard evaluations [18,19]. All the players were quite familiar with how to do the tests.

### 2.3. Procedures

#### 2.3.1. Anthropometric and Body Composition

All anthropometric and body composition measurements were performed during the morning [20], by a skilled person with 5 years of experience. Measurements were performed according to the international society for the advancement of kinanthropometry (ISAK) guidelines [21]. In order to measure height, sitting height, and weight, the participants stood without shoes and with just shorts. For measurement height parameters, the Seca model 213, Germany with an accuracy of ±5 mm and weight Seca model 813, UK with an accuracy of 0.1 per kg were used. Based on the information collected above and using the Mirwald formula, the maturity offset and age at PHV was determined [22]. The formula used is as follows: maturity offset = −9.236 + 0.0002708 (leg length × sitting height) − 0.001663 (age × leg length) + 0.007216 (age × sitting height) + 0.02292 (weight by height ratio), where R = 0.94, R2 = 0.891, and SEE = 0.592) and for leg length = standing height (cm) - sitting height (cm) was used.

To measure body fat percentage, seven subcutaneous fat thickness were used by the Jackson and Pollock method [22,23]. Data were collected by Lafayette Instrument Company (Lafayette, IN, USA) with an accuracy of 0.1 mm. All measurements were performed by one person on the right side of the body. The measured technical measurement error was considered according to the previous study [24].

#### 2.3.2. Monitoring Workloads Training

Each player was asked individually: “How did you feel about the intensity of the training?” for each session on a Category-Ratio-10 Borg scale, half an hour after training. In this scale, number one refers to a very easy training session and number ten refers to a very high-intensity training session [25]. The WL was calculated by multiplying the training time (minutes) with session RPE [10]. These players were familiar with this method during the previous two years in the team.

Other WL parameters were calculated as follows: a total load of daily training during the week was considered as weekly AWL; the uncoupled formula [16] was used to obtain the weekly CWL and ACWLR; weekly TM (weekly AWL ÷ SD of this week’s AWL); and eventually weekly TS (weekly AWL × weekly TM). This 20-week study was divided into 3 periods based on the competition schedule in the season then: EaS = W1 to W7; MiS = W8 to W13; and EnS = W14 to W20.

#### 2.3.3. The Modified 505 Test

The modified 505 test was used to assess COD [26]. This test was performed using a Newtest Power timer 300-series. The photocells of this device were adjusted based on each player’s hip height. After the warm-up, they stood at a distance of 70 cm before the start line. Immediately after hearing the sound of the starter, the player started to run inside the designated route, then passed through two photocells 5 m from the start line (midline). The time from here was recorded by the device. At this stage, the athlete crossed the finish line, which was 5 m away from the midline. It should be touched with one foot. Finally, the player quickly returned to the same route to cross the midline again. The photo-finish system was recorded at a time of complete (2 × 5 m). All subjects performed 2 trials test with a 3-min recovery. The best time of these two repetitions was considered as the record of each player. The intra-class correlation coefficient (ICC) was 0.94 for this test.

#### 2.3.4. Anaerobic Power Test

The RAST test was used to assess anaerobic power [27]. The settings of the photo-finish system were done with the players and how to start according to the modified 505 test. Each player performed 6 repetitions of 35 m between the photocells at maximum speed. There were only 10 s of rest between each repetition. Body mass was measured before the experiment. Then, based on the recorded times (each player in 6 repeats) after the test, the following formulas were used with Excel to obtain the results of anaerobic power variables; RAST of peak power (RPP) = the highest value; RAST of minimum power (RMP) = the lowest value; RAST of average power [28] = sum of all six values divided by 6; and RAST of fatigue index (RFI) = (RPP − RMP)/total time to covert the 6 sprints. The test retest ICC was 0.91 for this test.

### 2.4. Statistical Analysis

Statistical analyses were performed using GraphPad Prism 8.0.1 (GraphPad Software Inc, San Diego, California, USA). The significance level was set at *p* < 0.05. Data are presented as mean and SD. Shapiro–Wilk was applied to check the normality of the data. Pearson correlation analysis was performed between the WL parameters (except ACWLR) and RAP with PHV. While Spearman correlations were used for physical fitness tests (except RAP) and ACWLR, due to non-normality, this section has been done based on the mean differences between the steps. The effect size of the correlations was determined by considering the following thresholds [29,30]: <0.1 = trivial; 0.1–0.3 = small; > 0.3–0.5 = moderate; > 0.5–0.7 = large; > 0.7–0.9 = very large; and >0.9 = nearly perfect. Then, multiple linear regression analysis between training WL parameters, with variations in anaerobic power, COD, and maturity variables, were performed. The intended regression type was least-squares. The reliability for assessments, ICC were applied.

## 3. Results

Figure 1 shows weekly monitoring on training and matches load with the test timeline.

Descriptive characteristics of players are presented in Table 1. Values are reported as mean ± SD. In the whole season, the accumulated AWL was 31859 ± 1121 Arbitrary unit (A.U)., accumulated CWL was 28806 ± 995.1 A.U., accumulated ACWRL was 17.53 ± 0.3 A.U., accumulated TM was 23 ± 0.4 A.U., and ultimately, accumulated TS was 27821 ± 1075 A.U.

**Figure 2:**
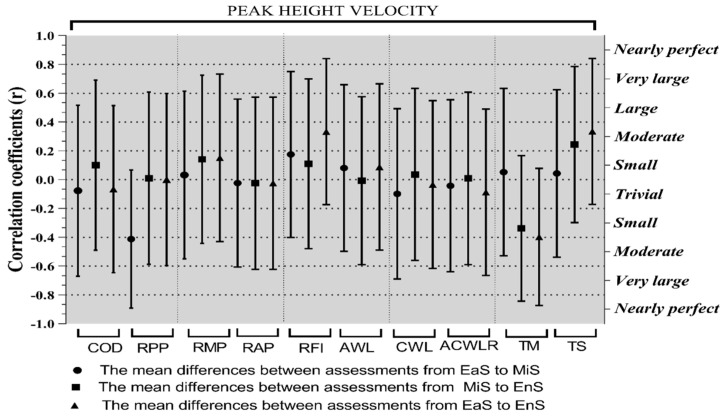
Correlation coefficients (at level of 95% confidence interval) for the peak height velocity soccer player. COD (seconds) = change of direction; RPP (watts) = Running-Based Anaerobic Sprint Test (RAST) of peak power; RMP (watts) = RAST of minimum power; RAP (watts)= RAST of average power; RFI = RAST of fatigue index; AWL(A.U.) = acute workload; CWL (A.U.) = chronic workload; ACWLR (A.U.) = acute: chronic workload ration; TM (A.U.) = training monotony; TS (A.U.) = training strain; and A.U. =Arbitrary unit.

Figure 2 shows the correlation coefficient between PHV with mean differences between fitness levels assessments and WL parameters between periods at the 95% confidence interval (EaS to MiS, and MiS to EnS, and EaS to EnS) and correlation coefficients at CI 95%. Based on the results, there were significant correlations between RPP at EaS to MiS (r = −0.48; CI 95% {−0.69 to 0.02}; *p* = 0.02), RFI at EaS to EnS (r = 0.39; CI 95% {−0.02 to 0.69}; *p* = 0.06), TM at MiS to EnS (r =−0.40; CI 95% {−0.02 to 0.69}; *p* = 0.06), TM at EaS to EnS (r=−0.46; CI 95% {−0.73 to −0.06}; *p* = 0.03); and TS at EaS to EnS (r = 0.39; CI 95% {−0.02 to 0.69}; *p* = 0.06) are moderate related to a PHV.

In the correlations between WL parameters with fitness variables, the most important of them were: RPP at EaS to MiS (r = 0.58 large; CI 95% {0.22 to 0.80}; *p* ≤ 0.001) and RMP at EaS to MiS (r = 0.42; CI 95% {0.013 to 0.71}; *p* = 0.04), which are largely and moderately related to AWL at EaS to MiS, respectively, also RPP at EaS to MiS (r = −0.55; CI 95% {−0.79 to 0.18}; *p* = 0.01) and RMP at EaS to MiS (r = −0.63; CI 95% {−0.83 to −0.29}; *p ≤* 0.001) are largely related to AWL at MiS to EnS. COD at EaS to EnS (r = 0.38; CI 95% {−0.034 to 0.69}; *p* = 0.07) and at MiS to EnS (r = 0.47; CI 95% {−0.07 to 0.73}; *p* = 0.02) are largely related to AWL at MiS to EnS. Additionally, COD at EaS to EnS (r = 0.38; CI 95% {−0.036 to 0.69}; *p* = 0.07) and at MiS to EnS (r = 0.47; CI 95% {0.067 to 0.74}; *p* = 0.02) are largely related to AWL at EaS to EnS.

In the CWL; COD at EaS to EnS (r = −0.41; CI 95% {−0.43 to 0.42}; *p* = 0.05), MiS to EnS (r = −0.39; CI 95% {−0.42 to 0.42}; *p* = 0.06), and EaS to MiS (r = −0.42; CI 95% {−0.45 to 0.39}; *p* = 0.05) are moderately related to CWL at MiS to EnS. Additionally, COD at EaS to EnS (r = −0.43; CI 95% {−0.71 to 0.02}; *p* = 0.04) and at MiS to EnS (r = −0.47; CI 95% {0.71 to 0.03}; *p* = 0.02) are moderately related, and RMP at EaS to MiS (r = 0.50; CI 95% {0.09 to 0.76}; *p* = 0.02) is largely related to CWL at EaS to EnS.

In the ACWLR; COD at EaS to EnS (r = 0.46; CI 95% {0.10 to 0.76}; *p* = 0.03), RPP at MiS to EnS (r = 0.44; CI 95% {0.025 to 0.73}; *p* = 0.04), and RAP at MiS to EnS (r = −0.42; CI 95% {−0.03 to 0.70}; *p* = 0.06) are moderately related to ACWLR at EaS to MiS and largely related with COD at MiS to EnS (r = 0.50; CI 95% {−0.29 to 0.53}; *p* = 0.01). Additionally, COD at EaS to EnS (r = 0.45; CI 95% {0.04 to 0.73}; *p* = 0.03) and at MiS to EnS (r = −0.58; CI 95% {0.22 to 0.80}; *p ≤* 0.001) are moderately and largely related to ACWLR at EaS to EnS, respectively (Table 2).

**Figure 3:**
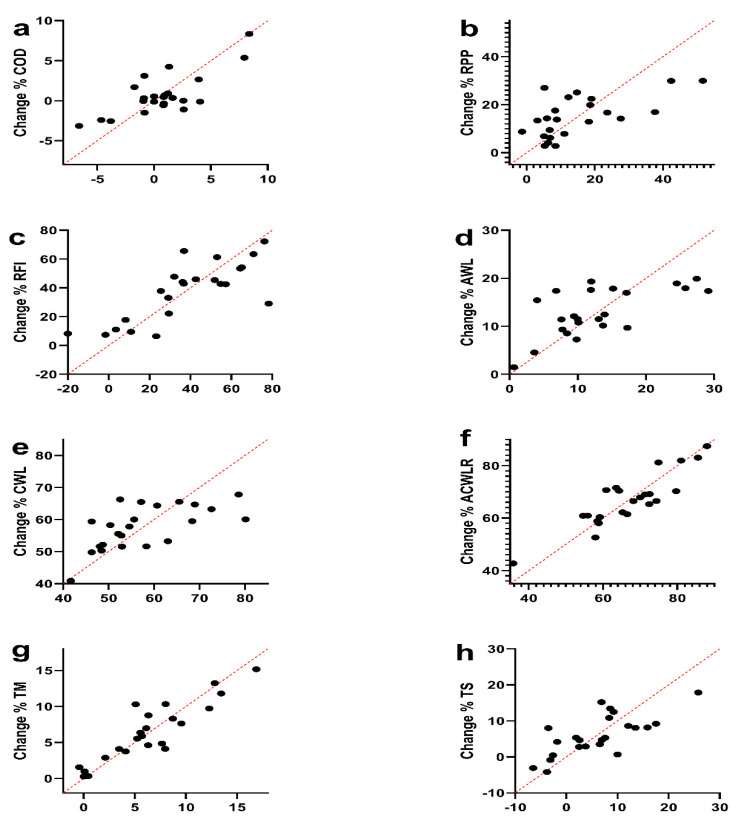
Multiple linear regression analysis was calculated to predict the percentage of change in fitness levels {i.e., COD (seconds), anaerobic power variables (watts), and workload parameter (A.U.)} based on accumulated workload parameters, baseline fitness levels, and the peak height velocity soccer player. (**a**); COD (seconds) = change of direction; (**b**); RPP (watts) = RAST of peak power; (**c**); RFI (%) = RAST of fatigue index; (**d**); AWL (A.U.) = acute workload; (**e**); CWL (A.U.) = chronic workload; (**f**); ACWLR (A.U.) = acute: chronic workload ration; (**g**); TM (A.U.) = training monotony; (**h**); TS (A.U.) = training strain; A.U. = Arbitrary unit.

Multiple linear regression analysis was calculated to predict the percentage of change in fitness levels {i.e., COD (seconds), anaerobic power variables (watts), and workload parameter (A.U.)} based on accumulated WL parameters, baseline fitness levels, and PHV soccer player (Table 3 and Figure 3). The first analysis in COD showed that there was no significance (*F*_(12, 10)_ = 1.17, *p* = 0.41), with *R^2^* of 0.58. Participants showed poor predictions for COD (Y) is equal to Beta0 + Beta1 (PHV) + Beta2 (Maturity offset) + Beta3 (EaS of COD) + Beta4 (EaS of RPP) + Beta5 (EaS of RMP) + Beta6 (EaS of RAP) + Beta7 (EaS of RFI) + Beta8 (EaS of AWL) + Beta9 (EaS of CWL) + Beta10 (EaS of ACWLR) + Beta11 (EaS of TM) + Beta12 (EaS of TS) where PHV and maturation status was coded or measured as years, fitness status was coded or measured as seconds, and watts and WL parameters were coded or measured as A.U. in order based on the equation. Additionally, the analysis in RPP and RFI demonstrated there was no significance *(F*_(12, 10)_ = 0.52, *p* = 0.86) and *F*_(12, 10)_ = 1.3, *p* = 0.36) with *R*^2^ of 0.38 and 0.60, respectively.

The second analysis in WL parameters {AWL (*F*_(12, 10)_ = 0.63, *P* = 0.78), with *R^2^* of 0.43 and CWL (*F*_(12, 10)_ = 0.59, *p* = 0.81), with *R^2^* of 0.41} showed that there was no significance for these variables. But there was significant ACWLR (*F*_(12, 10)_ = 2.93, *p* = 0.05), with *R^2^* of 0.78. participants showed poor predictions for WL parameters (Y) is equal to Beta0 + Beta1 (PHV) + Beta2 (Maturity offset) + Beta3 (EaS of COD) + Beta4 (EaS of RPP) + Beta5 (EaS of RMP) + Beta6 (EaS of RAP) + Beta7 (EaS of RFI) + Beta8 (EaS of AWL) + Beta9 (EaS of CWL) + Beta10 (EaS of ACWLR) + Beta11 (EaS of TM) + Beta12 (EaS of TS), where PHV and maturation status was coded or measured as years, fitness status was coded or measured as seconds, and watts and WL parameters were coded or measured as A.U. in order based on the equation. Participants’ predicted the ACWLR during the season decreased −0.048 A.U. for each A.U. of baseline CWL and −30.57 A.U. for each A.U. of baseline ACWLR. These variables were significant predictors of percentage of change in ACWLR levels during the competition season (Table 4). Among the variables of this study, it can be suggested that these two variables are the best predictors for the percentage of change of the players during a competition.

The third analysis in TM showed significance (*F*_(12, 10)_ = 3.49, *p* = 0.03), with a high *R^2^* of 0.81. Participants predicted TM (Y) is equal to Beta0 + Beta1 (PHV) + Beta2 (Maturity offset) + Beta3 (EaS of COD) + Beta4 (EaS of RPP) + Beta5 (EaS of RMP) + Beta6 (EaS of RAP) + Beta7 (EaS of RFI) + Beta8 (EaS of AWL) + Beta9 (EaS of CWL) + Beta10 (EaS of ACWLR) + Beta11 (EaS of TM) + Beta12 (EaS of TS), where PHV and maturation status was coded or measured as years, fitness status was coded or measured as seconds, and watts and WL parameters were coded or measured as A.U. in order based on the equation. Participants’ predicted TM during the season increased 10.47 A.U. for each year of PHV as well as decreased −0.1132 A.U. for each watts of RAP, and both PHV and baseline RAP were significant predictors of TM levels (Table 5). Therefore, among the variables of this study, it can be suggested that these two variables are the best predictors for the percentage of change of the players during a competition season. But there were no significant TS (*F*_(12, 10)_ = 0.89, *p* = 0.58), with an *R^2^* of 0.52.

## 4. Discussion

The first purpose of this research was to evaluate the relationship among the main differences between assessments and accumulated load parameters. Firstly, the accumulated daily WL during periods showed a large and a moderate correlation with RPP, COD, and RMP at EaS to MiS. To the best of our knowledge, this is the first study to investigate this chronic relationship in soccer players, using the internal WL calculated by the perceived exertion of the session. On the other hand, in a similar study that was done involving basketball athletes, Ferioli et al. found negative relationships between the volume training and changes in neuromuscular responses [31]. Similar negative influence of the WL on improvements in strength power was found in soccer, but the method employed to measure the internal training load was different from the perceived exertion method [32]. Different results among the mentioned studies and the present research could be associated with the specificity of each sport, periodization systems, and other training variables.

The main purpose of the internal WL control is to understand the magnitude of different works that are present in a training session, integrating both physiological and psychological parameters through the perceived exertion and time [33]. In spite of the fact that this practical method can be applied to identify the magnitude of different physical abilities [34,35], this lack of specificity could impair the use of this variable to predict the percentage of change in fitness level of anaerobic power and COD, which was confirmed by multiple linear regression analysis done in this research [36]. On the other hand, it can be suggested that the accumulated WL could predict the ACWLR and its percentage of change during a competition season, which is in agreement with other studies [36].

The ACWLR fatigue represents how much higher (ACWLR > 1.0) or lower (ACWLR < 1.0) the load of a specific week is, taking the internal WL of past microcyles into account. Originally, a value not higher than 1.5 was proposed in order to maintain a safe increase of the training WL [33], even though this value seems to be very relative depending on the sports, athletes, period of the season, and other variables [37]. Thus, Bowen et al. (2020) investigated the relationship between physical WL and injury risk in elite youth soccer players throughout two seasons and found that noncontact injury risk was associated with a high number of accelerations and with high acute high-speed distance combined with low chronic high-speed distances. Thus, it is clear that, in general, higher accumulated AWL can increase the injury risk, but, when this increase is followed by progressive increases in CWL, the players’ physical tolerance is increased as well [38,39].

Another commonly used variable to monitor the training program is the training monotony (TM) developed by Foster [40]. The present data showed that this variable can be predicted by the number of years the athlete is away from the PHV and by the average power identified by RAST test. The PHV is a minimally invasive, feasibly practical indicator of somatic maturation and it is related to the athletes who tend to be stronger, faster, and taller [41]. Therefore, it is safe to affirm that there is a strong relationship between the biological maturation and performance, which occurs due to the development of biological systems, such as cardiorespiratory and muscular systems. Philippaerts et al. (2005) evaluated balance, speed of limb movement, trunk strength, upper-body muscular endurance, explosive strength, running speed and COD, cardiorespiratory endurance, and anaerobic capacity and found that the peak development occurred at PHV.

To the best of our knowledge, this is the first study involving the analysis of the relationship between the total of all accumulated TM period and its relationship with the PHV. The results showed an increase in all of the accumulated TM of the period (10.5 U.A.) for each year of PHV. Monotony represents the variability among the WL and can be calculated through the average of daily training WL divided by SD of daily training WL. A previous study showed that values above 2.0 can represent an increase in the injury risk [40], which did not occur with the athletes analyzed in this research (average of TM was: 1.2 U.A.). Therefore, it is safe to affirm that the adequate values of monotony is related to optimal variability of the load, which is essential to maintain an appropriate increase of performance and reduction of injury risk as well [42]. However, it is difficult to compare the present data due to the low published researches providing reference values about this variable in young soccer athletes.

The relationship between the PHV and the accumulated TM of the period should be associated with the differences in CWL, even though there were no differences in changes of AWL between assessments from EaS to EnS. The point is that the CWL showed a positive association with the physical abilities. Thus, the higher physical capacity enables athletes to give a stronger effort, which could be expressed in the values of the chronic workload and the accumulated TM as well.

According to Clemente et al., the accumulated weekly training WL is largely correlated with performance parameters in professional soccer athletes, including the peak torque during knee extension. This fact can be associated with the bigger loads, which can be employed during the workouts, as the previously mentioned researches showed an increase in physical abilities, which followed the PHV [43,44].

One of the limitations of the present study could be the athletes’ levels of maturity. Although the method used has been validated (correlation coefficient = 0.83) [22], this is likely to change depending on the communities and the level of the athletes.

## 5. Conclusions

The current research reveals the associations between workload parameters and maturation and that some of the physical determinants can be influenced by concurrent qualities. This is important for possible talent identification processes and planning of the training.

## Figures and Tables

**Table 1:** Descriptive characteristics of 23 soccer player U16.

Variables	Mean ± SD	Confidence Interval 95%
Height (cm)	172.7 ± 4.2	171 to 174.4
Weight (kg)	61.3 ± 5.6	59 to 63.6
Sitting height (cm)	96.6 ± 2.1	91.8 to 93.4
Age at PHV (years)	13.6 ± 0.4	13.5 to 13.7
Maturity Offset (years)	1.9 ± 0.3	1.7 to 2
Age (years)	15.5 ± 0.2	15.3 to 15.5
Experience (years)	6.2 ± 1.6	5.6 to 6.9
VO_2max_ (mL.kg^−1^.min^−1^)	48.4 ± 2.6	47.3 to 49.4
Body Fat (%)	8.3 ± 2.9	7.2 to 9.5
AWL (A.U.)	31,859 ± 1121	31,374 to 32,344
CWL (A.U.)	28,806 ± 995.1	28,375 to 29,236
ACWLR (A.U.)	17.5 ± 0.3	17.4 to 17.7
TM (A.U.)	23 ± 0.4	22.8 to 23.2
TS (A.U.)	27,821 ± 1075	27,356 to 28,285

PHV = peak height velocity; VO_2max_ = maximal oxygen consumption; AWL = the accumulated acute workload in the season; CWL = the accumulated chronic workload in the season; ACWLR = the accumulated acute: chronic workload ration in the season; TM = the accumulated training monotony in the season; TS = the accumulated training strain in the season, and A.U. =Arbitrary unit.

**Table 2:** Correlation analysis between the workload parameters and physical fitness tests with peak height velocity.

Variable	β0	β1	β2	β3	β4	β5	β6	β7	β8	β9	β10	β11	β12	β13	β14	β15	β16	β17	β18	β19	β20	β21	β22	β23	β24	β25	β26	β27	β28	β29	β30
PHV(β0)	1.00																														
COD1(β1)	−0.09	1.00																													
COD2(β2)	0.12	**0.41**	1.00																												
COD3(β3)	−0.08	0.83	**0.77**	1.00																											
RPP1(β4)	**−0.48**	0.25	−0.34	−0.21	1.00																										
RPP2(β5)	0.01	−0.09	0.02	0.02	−0.08	1.00																									
RPP3(β6)	0.00	−0.03	−0.15	−0.10	0.14	**0.93**	1.00																								
RMP1(β7)	0.04	−0.30	−0.29	−0.37	**0.58**	0.11	0.18	1.00																							
RMP2(β8)	0.17	0.05	0.05	0.05	0.14	**0.47**	**0.46**	0.13	1.00																						
RMP3(β9)	0.18	0.04	0.06	0.03	0.12	**0.45**	**0.46**	0.24	**0.97**	1.00																					
RAP1(β10)	−0.03	−0.15	−0.21	−0.22	0.26	0.12	0.15	**0.49**	0.22	0.26	1.00																				
RAP2(β11)	−0.03	0.15	0.08	0.16	0.08	**0.79**	**0.75**	0.09	**0.53**	**0.53**	−0.09	1.00																			
RAP3(β12)	−0.03	0.16	0.06	0.13	0.15	**0.73**	**0.73**	0.23	**0.47**	**0.50**	0.16	**0.93**	1.00																		
RFI1(β13)	0.21	0.14	0.26	0.29	−0.29	0.17	0.09	−0.36	0.27	0.22	−0.04	0.27	0.24	1.00																	
RFI2(β14)	0.13	0.03	0.21	0.04	0.14	**−0.51**	**−0.51**	0.20	−0.07	−0.02	0.02	−0.21	−0.17	−0.25	1.00																
RFI3(β15)	**0.39**	−0.16	0.33	0.09	−0.09	**−0.52**	**−0.53**	−0.06	0.34	0.33	−0.10	−0.13	−0.13	0.25	**0.91**	1.00															
AWL1(β16)	0.10	−0.04	−0.12	−0.10	**0.58**	−0.01	0.07	**0.42**	0.16	0.19	0.06	0.01	0.01	0.02	0.05	0.05	1.00														
AWL2(β17)	−0.01	0.17	**0.47**	0.38	**−0.55**	−0.06	−0.13	**−0.63**	−0.04	−0.10	−0.28	−0.01	−0.04	0.26	0.09	0.20	**−0.69**	1.00													
AWL3(β18)	0.11	0.17	**0.47**	0.38	−0.03	−0.08	−0.08	−0.31	0.14	0.11	−0.29	0.00	−0.04	0.36	0.17	0.32	0.30	**0.48**	1.00												
CWL1(β19)	−0.12	−0.04	0.00	−0.01	−0.21	0.02	−0.09	−0.20	−0.05	−0.10	−0.04	−0.02	−0.09	−0.12	0.02	0.12	**−0.80**	**0.50**	−0.26	1.00											
CWL2(β20)	0.04	**−0.42**	**−0.39**	**−0.41**	0.26	0.09	0.22	**0.50**	0.14	0.19	0.26	0.13	0.24	−0.07	−0.05	−0.27	0.35	**−0.78**	**−0.53**	**−0.62**	1.00										
CWL3(β21)	−0.04	−0.24	**−0.47**	**−0.43**	0.01	0.09	0.09	0.31	−0.04	−0.01	0.35	0.05	0.09	−0.35	−0.08	−0.22	−0.33	**−0.42**	**−0.94**	0.22	0.23	1.00									
ACWLR1(β22)	−0.05	0.15	**0.50**	**0.46**	−0.12	**0.44**	0.33	−0.22	0.11	0.13	−0.34	**0.40**	0.33	0.25	−0.13	−0.09	−0.04	0.20	0.28	−0.01	−0.18	−0.24	1.00								
ACWLR2(β23)	0.01	−0.25	−0.32	−0.31	0.09	−0.31	−0.25	0.02	−0.25	−0.34	0.25	−0.21	−0.21	−0.05	0.08	0.23	−0.18	0.23	−0.08	0.20	0.02	0.25	**−0.63**	1.00							
ACWLR3(β24)	−0.11	0.16	**0.58**	**0.45**	−0.33	0.12	0.08	−0.35	−0.02	−0.05	−0.20	0.15	0.13	0.23	−0.06	0.04	−0.38	**0.53**	0.25	0.20	−0.25	−0.11	**0.49**	0.08	1.00						
TM1(β25)	0.06	−0.05	−0.16	−0.12	0.13	−0.14	−0.12	0.23	−0.34	−0.31	−0.23	−0.31	−0.33	0.18	−0.04	0.03	0.20	−0.29	−0.13	0.05	−0.01	0.11	−0.08	0.13	0.06	1.00					
TM2(β26)	**−0.40**	−0.09	−0.01	−0.06	−0.30	0.17	0.14	−0.29	0.10	0.07	0.20	0.24	0.26	0.02	−0.11	−0.10	−0.31	**0.46**	0.23	0.02	−0.09	−0.15	0.06	0.04	0.18	**−0.63**	1.00				
TM3(β27)	**−0.46**	−0.15	−0.13	−0.17	−0.29	0.12	0.08	−0.20	−0.13	−0.15	0.08	0.07	0.08	0.17	−0.17	−0.10	−0.25	0.38	0.19	0.07	−0.12	−0.12	0.02	0.15	0.28	−0.05	**0.81**	1.00			
TS1(β28)	0.05	−0.05	−0.10	−0.09	0.52	0.06	0.12	0.35	0.29	0.32	0.16	0.12	0.14	−0.08	0.07	0.03	**0.91**	**−0.57**	0.34	**−0.45**	0.29	−0.36	−0.11	−0.16	**−0.45**	−0.21	−0.02	−0.19	1.00		
TS2(β29)	0.29	0.26	**0.50**	**0.46**	**−0.41**	−0.19	−0.24	**−0.50**	−0.13	−0.17	**−0.43**	−0.18	−0.23	0.30	0.15	0.27	**−0.54**	**0.76**	0.35	0.34	**−0.48**	−0.32	0.17	0.10	**0.46**	0.14	−0.22	−0.17	**−0.63**	1.00	
TS3(β30)	**0.39**	0.24	**0.46**	**0.42**	0.14	−0.15	−0.13	−0.16	0.20	0.18	−0.31	−0.06	−0.10	0.25	0.25	0.35	**0.45**	0.20	**0.80**	−0.13	−0.22	**−0.79**	0.06	−0.07	0.00	−0.09	−0.28	**−0.42**	**0.45**	**0.41**	1.00

Significant differences (*p* ≤ 0.05) are highlighted in bold. PHV = Peak height velocity; COD = change of direction; RPP = Running-Based Anaerobic Sprint Test (RAST) of peak power; RMP = RAST of minimum power; RAP = RAST of average power; RFI = RAST of fatigue index; AWL = acute workload; CWL = chronic workload; ACWLR = acute: chronic workload ration; TM = training monotony; and TS = training strain; and 1, 2, and 3 = the mean differences between assessments (EaS to MiS, and MiS to EnS, and EaS to EnS), respectively.

**Table 3:** Multiple linear regression analysis: percentage of change between EaS and EnS in COD and anaerobic power variables with workload parameters, baseline fitness levels, and PHV.

**Variable**	**Beta**	**Estimate**	**|t|**	***p* Value**	**95% CI for Estimated**	
COD (%)	β0	98.47	0.40	0.70	−453 to 649.9	***R***^2^ = 0.58
PHV (years)	β1	1.82	0.38	0.710	−8.80 to 12.45	
Maturity offset (years)	β2	7.87	1.16	0.273	−7.25 to 22.99	
COD (Seconds)	β3	8.78	1.24	0.245	−7.06 to 24.62	
RPP (watts)	β4	−0.15	1.66	0.129	−0.36 to 0.05	
RMP (watts)	β5	0.13	1.20	0.259	−0.11 to 0.36	
RAP (watts)	β6	0.01	0.34	0.742	−0.08 to 0.11	
RFI	β7	4.15	1.4	0.192	−2.46 to 10.76	
AWL (A.U.)	β8	0.01	0.83	0.424	−0.02 to 0.032	
CWL (A.U.)	β9	−0.01	0.83	0.423	−0.02 to 0.01	
ACWLR (A.U.)	β10	−6.02	1.71	0.119	−13.9 to 1.84	
TM (A.U.)	β11	−2.86	0.28	0.789	−26.08 to 20.35	
TS (A.U.)	β12	−0.004	0.44	0.671	−0.02 to 0.02	
**Variable**	**Beta**	**Estimate**	**|t|**	***p* value**	**95% CI for Estimated**	
RPP (%)	β0	−522	0.45	0.668	−3156 to 2112	***R***^2^ = 0.38
PHV (years)	β1	13.71	0.60	0.561	−37.05 to 64.47	
Maturity offset (years)	β2	34.71	1.07	0.309	−37.53 to 106.9	
COD (Seconds)	β3	7.26	0.21	0.835	−68.39 to 82.91	
RPP (watts)	β4	−0.11	0.25	0.809	−1.10 to 0.88	
RMP (watts)	β5	−0.06	0.11	0.911	−1.18 to 1.06	
RAP (watts)	β6	0.15	0.72	0.491	−0.30 to 0.59	
RFI	β7	0.08	0.01	0.996	−31.49 to 31.64	
AWL (A.U.)	β8	−0.01	0.2	0.848	−0.12 to 0.10	
CWL (A.U.)	β9	0.06	0.51	0.624	−0.052 to 0.08	
ACWLR (A.U.)	β10	−2.85	0.17	0.869	−40.42 to 34.72	
TM (A.U.)	β11	8.04	0.16	0.875	−102.9 to 118.9	
TS (A.U.)	β12	0.001	0.03	0.976	−0.09 to 0.09	
**Variable**	**Beta**	**Estimate**	**|t|**	***p* value**	**95% CI for Estimated**	
RFI (%)	β0	−141.9	0.08	0.94	−4213 to 3929	***R***^2^ = 0.60
PHV (years)	β1	−45.26	1.29	0.23	−123.7 to 33.19	
Maturity offset (years)	β2	−30.31	0.61	0.559	−141.9 to 81.33	
COD (Seconds)	β3	−87.38	1.67	0.127	−204.3 to 29.54	
RPP (watts)	β4	0.59	0.85	0.415	−0.95 to 2.12	
RMP (watts)	β5	−0.55	0.70	0.498	−2.27 to 1.18	
RAP (watts)	β6	0.02	0.06	0.957	−0.68 to 0.71	
RFI	β7	−21.33	0.97	0.353	−70.12 to 27.45	
AWL (A.U.)	β8	−0.02	0.21	0.840	−0.19 to 0.16	
CWL (A.U.)	β9	−0.02	0.45	0.665	−0.12 to 0.08	
ACWLR (A.U.)	β10	15.03	0.58	0.577	−43.02 to 73.09	
TM (A.U.)	β11	35.21	0.46	0.657	−136.2 to 206.6	
TS (A.U.)	β12	0.04	0.63	0.546	−0.10 to 0.18	

*β0* = Y; EaS = early-season; EnS = end-season; PHV = peak height velocity; COD = change of direction; RPP = Running-Based Anaerobic Sprint Test (RAST) of peak power; RMP = RAST of minimum power; RAP = RAST of average power; RFI = RAST of fatigue index; AWL = acute workload; CWL = chronic workload; ACWLR = acute: chronic workload ration; TM = training monotony; TS = training strain; % = the percentage of change in between assessments from EaS to EnS; and CI = confidence interval.

**Table 4:** Multiple linear regression analysis: percentage of change between EaS and EnS in AWL, CWL, and ACWLR with workload parameters, baseline fitness levels, and PHV.

**Variable**	**Beta**	**Estimate**	**|t|**	***p* Value**	**95% CI for Estimated**	
AWL (%)	β0	−500	0.78	0.455	−1933 to 932.6	***R***^2^ = 0.43
PHV (years)	β1	6.69	0.54	0.601	−20.92 to 34.29	
Maturity offset (years)	β2	17.29	0.98	0.350	−22 to 56.58	
COD (Seconds)	β3	5.15	0.28	0.786	−36 to 46.29	
RPP (watts)	β4	−0.12	0.49	0.636	−0.66 to 0.42	
RMP (watts)	β5	0.21	0.76	0.467	−0.40 to 0.81	
RAP (watts)	β6	−0.09	0.79	0.446	−0.33 to 0.16	
RFI	β7	4.14	0.54	0.603	−13.03 to 21.31	
AWL (A.U.)	β8	0.00	0.14	0.889	−0.07 to 0.06	
CWL (A.U.)	β9	0.00	0.06	0.957	−0.04 to 0.04	
ACWLR (A.U.)	β10	4.70	0.51	0.619	−15.73 to 25.13	
TM (A.U.)	β11	11.66	0.43	0.676	−48.65 to 71.97	
TS (A.U.)	β12	0.01	0.30	0.771	−0.04 to 0.06	
**Variable**	**Beta**	**Estimate**	**|t|**	***p* value**	**95% CI for Estimated**	
CWL (%)	β0	−510.80	0.57	0.583	−2519 to 1497	***R***^2^ = 0.41
PHV (years)	β1	13.46	0.78	0.456	−25.23 to 52.16	
Maturity offset (years)	β2	28.53	1.15	0.275	−26.54 to 83.60	
COD (Seconds)	β3	17.76	0.69	0.508	−39.91 to 75.43	
RPP (watts)	β4	−0.35	1.02	0.332	−1.10 to 0.41	
RMP (watts)	β5	0.48	1.25	0.241	−0.38 to 1.33	
RAP (watts)	β6	−0.15	0.95	0.366	−0.49 to 0.20	
RFI (%)	β7	10.89	1.01	0.337	−13.17 to 34.95	
AWL (A.U.)	β8	−0.01	0.33	0.749	−0.098 to 0.07	
CWL (A.U.)	β9	0.01	0.44	0.670	−0.04 to 0.06	
ACWLR (A.U.)	β10	1.43	0.11	0.914	−27.21 to 30.06	
TM (A.U.)	β11	11.41	0.30	0.770	−73.11 to 95.94	
TS (A.U.)	β12	0.01	0.16	0.875	−0.07 to 0.08	
**Variable**	**Beta**	**Estimate**	**|t|**	***p* value**	**95% CI for Estimated**	
ACWLR (%)	β0	442.60	0.73	0.481	−904.8 to 1790	***R****^2^*= *0.78*
PHV (years)	β1	1.39	0.12	0.908	−24.58 to 27.36	
Maturity offset (years)	β2	8.93	0.54	0.602	−28.02 to 45.89	
COD (Seconds)	β3	−0.71	0.04	0.968	−39.41 to 37.99	
RPP (watts)	β4	0.18	0.80	0.445	−0.33 to 0.69	
RMP (watts)	β5	−0.13	0.49	0.636	−0.68 to 0.45	
RAP (watts)	β6	−0.01	0.13	0.896	−0.24 to 0.22	
RFI (%)	β7	−4.97	0.69	0.508	−21.12 to 11.18	
AWL (A.U.)	β8	0.04	1.75	0.111	−0.01 to 0.10	
CWL (A.U.)	β9	−0.05	3.12	**0.011**	−0.08 to −0.01	
ACWLR (A.U.)	β10	−30.57	3.54	**0.005**	−49.79 to −11.35	
TM (A.U.)	β11	1.50	0.06	0.954	−55.23 to 58.22	
TS (A.U.)	β12	≤ 0001	0.02	0.981	−0.05 to 0.05	

Significant differences (*p* ≤ 0.05) are highlighted in bold. β0 = Y; EaS = early-season; EnS = end-season; PHV = Peak height velocity; COD = change of direction; RPP = RAST of peak power; RMP = RAST of minimum power; RAP = RAST of average power; RFI = RAST of fatigue index; AWL = acute workload; CWL = chronic workload; ACWLR = acute: chronic workload ration; TM = training monotony; TS = training strain; %= the percentage of change in between assessments from EaS to EnS; and CI = confidence interval.

**Table 5:** Multiple linear regression analysis: percentage of change between EaS and EnS in TM and TS with workload parameters, baseline fitness levels, and PHV.

**Variable**	**Beta**	**Estimate**	**|t|**	***p* Value**	**95% CI for Estimated**	
TM (%)	β0	−220.4	0.99	0.345	−716.3 to 275.5	***R***^2^ = 0.81
PHV (years)	β1	10.5	2.44	**0.035**	0.91 to 20.02	
Maturity offset (years)	β2	8.7	1.43	0.184	−4.89 to 22.31	
COD (Seconds)	β3	1.5	0.23	0.826	−12.80 to 15.69	
RPP (watts)	β4	≤0001	0.02	0.988	−0.19 to 0.19	
RMP (watts)	β5	0.1	1.07	0.309	−0.11 to 0.31	
RAP (watts)	β6	−0.11	2.99	**0.014**	−0.19 to −0.03	
RFI (%)	β7	1.28	0.48	0.641	−4.66 to 7.2	
AWL (A.U.)	β8	≤0001	0.38	0.715	−0.02 to 0.03	
CWL (A.U.)	β9	−0.01	1.24	0.245	−0.02 to 0.01	
ACWLR (A.U.)	β10	−2.44	0.77	0.460	−9.51 to 4.63	
TM (A.U.)	β11	2.85	0.30	0.768	−18.03 to 23.72	
TS (A.U.)	β12	≤0001	0.64	0.538	−0.01 to 0.02	
**Variable**	**Beta**	**Estimate**	**|t|**	***p* value**	**95% CI for Estimated**	
TS (%)	β0	−144.80	0.24	0.817	−1504 to 1215	***R***^2^ = 0.52
PHV (years)	β1	−4.41	0.38	0.715	−30.62 to 21.79	
Maturity offset (years)	β2	7.53	0.45	0.663	−29.76 to 44.81	
COD (Seconds)	β3	1.91	0.11	0.916	−37.14 to 40.96	
RPP (watts)	β4	−0.11	0.48	0.643	−0.62 to 0.40	
RMP (watts)	β5	0.08	0.32	0.758	−0.49 to 0.66	
RAP (watts)	β6	0.04	0.35	0.735	−0.19 to 0.27	
RFI (%)	β7	2.52	0.34	0.738	−13.78 to 18.81	
AWL (A.U.)	β8	≤0001	0.11	0.914	−0.07 to 0.06	
CWL (A.U.)	β9	0.01	0.35	0.733	−0.03 to 0.04	
ACWLR (A.U.)	β10	8.67	1.00	0.343	−10.72 to 28.06	
TM (A.U.)	β11	2.41	0.09	0.927	−54.83 to 59.65	
TS (A.U.)	β12	≤0001	0.13	0.896	−0.05 to 0.05	

Significant differences (*p* ≤ 0.05) are highlighted in bold. β0 = Y; EaS = early-season; EnS = end-season; PHV = Peak height velocity; COD = change of direction; RPP = RAST of peak power; RMP = RAST of minimum power; RAP = RAST of average power; RFI = RAST of fatigue index; AWL = acute workload; CWL = chronic workload; ACWLR = acute: chronic workload ration; TM = training monotony; and TS = training strain; % = the percentage of change in between assessments from EaS to EnS; and CI = confidence interval.
